# Data normalization for addressing the challenges in the analysis of single-cell transcriptomic datasets

**DOI:** 10.1186/s12864-024-10364-5

**Published:** 2024-05-06

**Authors:** Raquel Cuevas-Diaz Duran, Haichao Wei, Jiaqian Wu

**Affiliations:** 1 Tecnologico de Monterrey, Escuela de Medicina y Ciencias de la Salud, Monterrey, Nuevo Leon, 64710 Mexico; 2 The Vivian L. Smith Department of Neurosurgery, McGovern Medical School, The University of Texas Health Science Center at Houston, Houston, TX 77030 USA; 3 Center for Stem Cell and Regenerative Medicine, UT Brown Foundation Institute of Molecular Medicine, Houston, TX 77030 USA; 4 MD Anderson Cancer Center UTHealth Graduate School of Biomedical Sciences, Houston, TX 77030 USA

**Keywords:** Single-cell sequencing, scRNA-seq, Normalization, Technical variability, Biological variability

## Abstract

**Background:**

Normalization is a critical step in the analysis of single-cell RNA-sequencing (scRNA-seq) datasets. Its main goal is to make gene counts comparable within and between cells. To do so, normalization methods must account for technical and biological variability. Numerous normalization methods have been developed addressing different sources of dispersion and making specific assumptions about the count data.

**Main body:**

The selection of a normalization method has a direct impact on downstream analysis, for example differential gene expression and cluster identification. Thus, the objective of this review is to guide the reader in making an informed decision on the most appropriate normalization method to use. To this aim, we first give an overview of the different single cell sequencing platforms and methods commonly used including isolation and library preparation protocols. Next, we discuss the inherent sources of variability of scRNA-seq datasets. We describe the categories of normalization methods and include examples of each. We also delineate imputation and batch-effect correction methods. Furthermore, we describe data-driven metrics commonly used to evaluate the performance of normalization methods. We also discuss common scRNA-seq methods and toolkits used for integrated data analysis.

**Conclusions:**

According to the correction performed, normalization methods can be broadly classified as within and between-sample algorithms. Moreover, with respect to the mathematical model used, normalization methods can further be classified into: global scaling methods, generalized linear models, mixed methods, and machine learning-based methods. Each of these methods depict pros and cons and make different statistical assumptions. However, there is no better performing normalization method. Instead, metrics such as silhouette width, K-nearest neighbor batch-effect test, or Highly Variable Genes are recommended to assess the performance of normalization methods.

**Supplementary Information:**

The online version contains supplementary material available at 10.1186/s12864-024-10364-5.

## Background

Single-cell RNA-sequencing (scRNA-seq) has become a powerful approach to simultaneously quantify the transcription of hundreds or even thousands of features (genes, transcripts, exons) at an unprecedented resolution. This high-throughput transcriptomic profiling assays have helped scientists to study important biological questions, for example, cellular heterogeneity, dynamics of cellular processes and pathways, novel cell type discovery, and cell fate decisions and differentiation [1–4].

While the expression matrices obtained from bulk RNA-seq are structurally very similar to those derived from scRNA-seq experiments, there are distinct features that characterize scRNA-seq datasets mainly due to the scarcity of starting material and the high resolution. These features include an unusually high abundance of zeros, an increased cell-to-cell variability, and complex expression distributions. This high intercellular variability of read counts or overdispersion is derived from biological and technical factors [5]. Understanding the contribution of each of these factors to the global dispersion is important since technical variability can be confounded by biological differences. Thus, statistical and computational methods used for analyzing scRNA-seq datasets face the challenge of separating wanted from unwanted variation.

Many normalization methods exist for bulk RNA-seq and have been applied to scRNA-seq. However, the specific features of scRNA-seq datasets have triggered the development of specific normalization strategies. Herein, we briefly describe the commonly used methods for scRNA-seq, including isolation and library preparation protocols. We also discuss the causes and effects of technical and biological sources of variability, focusing mainly on those derived from measurement inefficiencies. Next, we summarize state-of-the-art normalization methods, incorporating those that have been specifically tailored to scRNA-seq datasets. We also delineate imputation and batch-effect correction methods. Furthermore, we describe data-driven metrics that are commonly used to evaluate the performance of normalization methods. Finally, we highlight commonly used toolkits and provide practical recommendations for scRNA-seq users.

## Main text

### Single-cell RNA-sequencing methods

The first step in a scRNA-seq experiment is the preparation of a high-quality single-cell suspension. Single-nuclei can also be isolated, however, for simplicity we will refer to both as single-cells. The condition of the cells isolated is critical for a successful experiment. Isolation methods can expose cells to harsh enzymatic methods or chemical conditions that can stress cells and generate unwanted variations in gene expression [6]. Single-cells can be isolated from suspensions (e.g. blood) or from solid tissues (e.g. tumor). Samples can be obtained from fresh (e.g. resection surgeries, cell cultures) or preserved sources (e.g. postmortem brains). The protocols for preparing cell suspensions depend on the source of cells and pilot experiments may be required to ensure the optimal condition of cells.

#### Isolating single-cells

**Fig. 1:**
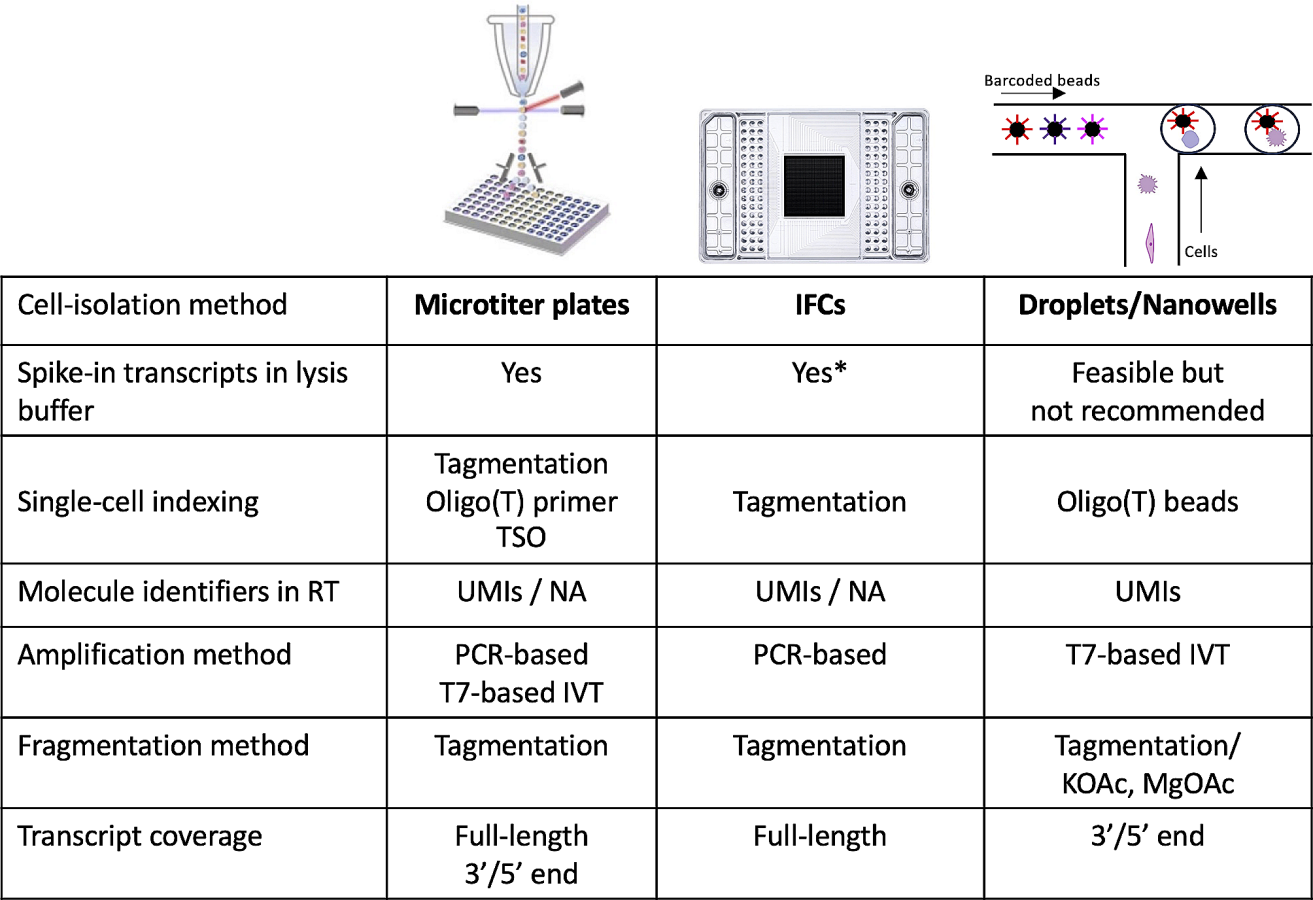
Overview of common scRNA-seq workflows and their characteristics. *only in cases where the volumes to be added for each reagent can be modified and don’t depend on the design of the reaction chamber. IFC = integrated fluidics circuits, RT = reverse transcription, TSO = template-switching oligonucleotide, UMI = unique molecular identifiers, PCR = polymerase chain reaction, IVT = in vitro transcription, NA = not available, KOAc = potassium acetate, MgOAc = magnesium acetate. Figure in droplets/nanowells column was adapted from [12]

Cells within the suspension need to be isolated or captured to obtain individual reaction volumes. To date, numerous isolation methods have been used including manual methods (serial limited dilution, microdissection or pipetting [7]) and automated technologies (fluorescence/magnetic-activated cell sorting (FACS/MACS) [8, 9] or microfluidics [10]). Depending on the research question, certain applications are better suited for cell isolation. For example, profiling of cancer cells requires the exclusion of blood cells, thus FACS or MACS may be used to filter the cellular suspension. Applications in which an unbiased view of the cellular composition is desired do not require filtering. In this case the cellular suspension can be directly used as input in a microfluidics system in an adequate dilution. The three most common workflows used to isolate single cells are microtiter plates, microfluidics, and droplets/nanowells, as shown in Fig. 1. See Additional file 1 for an extended list of methods and characteristics.

The most representative example of microplate-based cell-isolation methods is FACS. FACS sorts cells into wells or microtiter plates where they are ready for manual or automated library preparation. The advantage of this method is that it allows the exclusion of dead or damaged cells and the enrichment of cells depicting specific antibody-labelled proteins. Furthermore, microplates can be imaged to ensure that no doublets or empty wells are present. Reagents required for lysis and library preparation are then added to each well. Microfluidics based cell-isolation methods include the use of integrated fluidics circuits (IFCs), typically the Fluidigm C1 System. An IFC consists of a chip with miniature lanes that contain traps. The cellular suspension flows through the chip and cells are caught in each trap. Then, reagents for lysis and library preparation flow through the chip and cells are processed in consecutive nanoliter reaction chambers. Another method also uses a microfluidic system but instead of using traps, it encapsulates cells in droplets or captures them in nanowells. Droplet-based systems use a water-in-oil emulsion to encapsulate single-cells. This drop of emulsion contains reagents for RT (reverse transcription) as well as randomly introduced barcodes for tagging cells. Common droplet-based platforms include inDrops [11], Drop-Seq [12] and 10X Genomics [13]. In nanowell platforms cells are loaded into nanowells with pre-loaded barcoded beads. A typical example of this platform is Seq-Well [14].

#### Capturing mRNA molecules and generating cDNA

Once the cells are isolated, they are lysed and exogenous spike-in RNA molecules, for example the External RNA Control Consortium (ERCCs) spike-ins [15], may be added. Spike-in RNAs are used to create a standard baseline measurement for counting and normalization [16]. As will be described in the next section, the addition of spike-ins is not feasible for all platforms. After cell lysis, poly(A)-tailed mRNA is captured by poly(T) oligonucleotides and then reverse transcribed into cDNA. Importantly, the poly(T) oligonucleotides may include single-cell-specific barcodes for cell identification and a random nucleotide sequence that will be used as a unique molecule identifier (UMI). UMIs are used for efficiently counting mRNA molecules and correcting PCR-induced artifacts [17] as will be described in the next section.

#### Amplifying cDNA

After RT, cDNA is amplified typically by PCR or T7-based in vitro transcription (IVT). PCR amplification is commonly performed using two methods: Tang protocol and template-switching oligonucleotides (TSO). In the Tang protocol [18], mRNAs are reverse transcribed into cDNA using poly(T) primers with an anchor sequence (UP1). Then poly(A) tails are added to the 3’ ends of cDNAs, and second strands are synthesized using poly(T) primers with another anchor (UP2). Finally, cDNAs are PCR-amplified using both anchors. In the TSO protocol, the reverse transcriptase adds cytosines to the cDNA allowing the template switching reaction and the addition of PCR adaptor sequences. Variants of the TSO protocol are implemented in single-cell tagged reverse transcription sequencing (STRT-seq) [19], switching mechanisms at the 5’-end of the RNA transcript sequencing (Smart-seq) [20], and Smart-seq2 [21]. These sequencing protocols can be performed in the microtiter plate and IFC platforms in combination with tagmentation methods for sequencing library preparation. Tagmentation involves using an enzyme that simultaneous generates fragments and adds cell indexes.

PCR-based methods are exponential and non-linear amplification techniques. They are more efficient than IVT methods, however both introduce technical biases as will be described. IVT requires the addition of a T7 promoter in the poly(T) primer and it doesn’t require template switching. Numerous platforms use T7-based IVT amplification, for example cell expression by linear amplification and sequencing (CEL-seq) [7], CEL-seq2 [22], massively parallel single-cell RNA sequencing (MARS-seq) [23], and indexing droplets RNA sequencing (inDrops-seq) [24]. Amplified cDNA or RNA (PCR or IVT) is fragmented during library preparation and adaptors are added. Different fragmentation methods can be used, for example, tagmentation or mechanical fragmentation.

#### Transcript coverage

An important consideration is the transcript coverage when selecting the scRNA-seq protocol. Expression profiling of single-cells can be done by sequencing full-length transcripts or by merely counting 3’ or 5’ molecule ends, referred to as digital counting (see Fig. 1 and Additional file 1). Full-length scRNA-seq protocols offers several advantages, for example, the detection of low-expressed transcripts [25], splice variants and isoforms, single-nucleotide variants [26, 27], and fusion transcripts [28]. However, full-length sequencing methods are limited by lower cellular throughputs and higher costs [29]. Moreover, until recently, commercial plate-based full-length sequencing protocols did not incorporate UMIs [30]. Novel full-length sequencing methods now integrate UMI’s in the TSO sequence increasing transcript quantification accuracy. Examples of these methods include Smart-Seq3 [31], Smart-seqxpress [32], and Flash-seq [33]. Another disadvantage of full-length protocols is that they do not allow early cell barcoding and thus, pooling can’t be performed. Droplet-based methods rely on digital counting, representing a cost-effective alternative. However, since these methods sequence only a small fragment of the 3’ or 5’ end of transcripts, isoform identification becomes highly challenging [34, 35]. Methods for quantifying isoforms from 3’ droplet-based assays (e.g. 10X Genomics) are emerging. For example, Scasa [35], a method that estimates isoform expression based on transcription clusters and isoform paralogs, and STARsolo [36], a mapping/quantification tool that has been used to quantify splicing events in 3’ droplet-based datasets.

#### General scRNA-seq approaches

Overall, there are two common approaches to scRNA-seq: isolating a large number of cells and sequencing libraries in a low depth (e.g. droplet-based) or isolating fewer cells and implementing a higher sequencing depth (e.g. microplate-based). Detailed descriptions of each platform have been reviewed in [25, 37–39]. A prominent multicenter benchmarking study was performed to evaluate the performance of 13 commonly used scRNA-seq protocols including plate-based methods and microfluidic systems (droplets, nanowells, and IFC) [29]. In this study, a complex reference sample (high cell-type heterogeneity, closely related subpopulations, known cell composition and cell markers) was used to compare the capability of these protocols in describing tissue complexity [29]. Authors demonstrated differences among the protocols in library complexity and in their ability to detect cell subpopulation markers. Therefore, users should make informed decisions when designing a single-cell RNA-seq study to detect an adequate number and complexity of RNA molecules that can predict the cell phenotypes and infer their function.

### The challenges of single-cell datasets

**Fig. 2:**
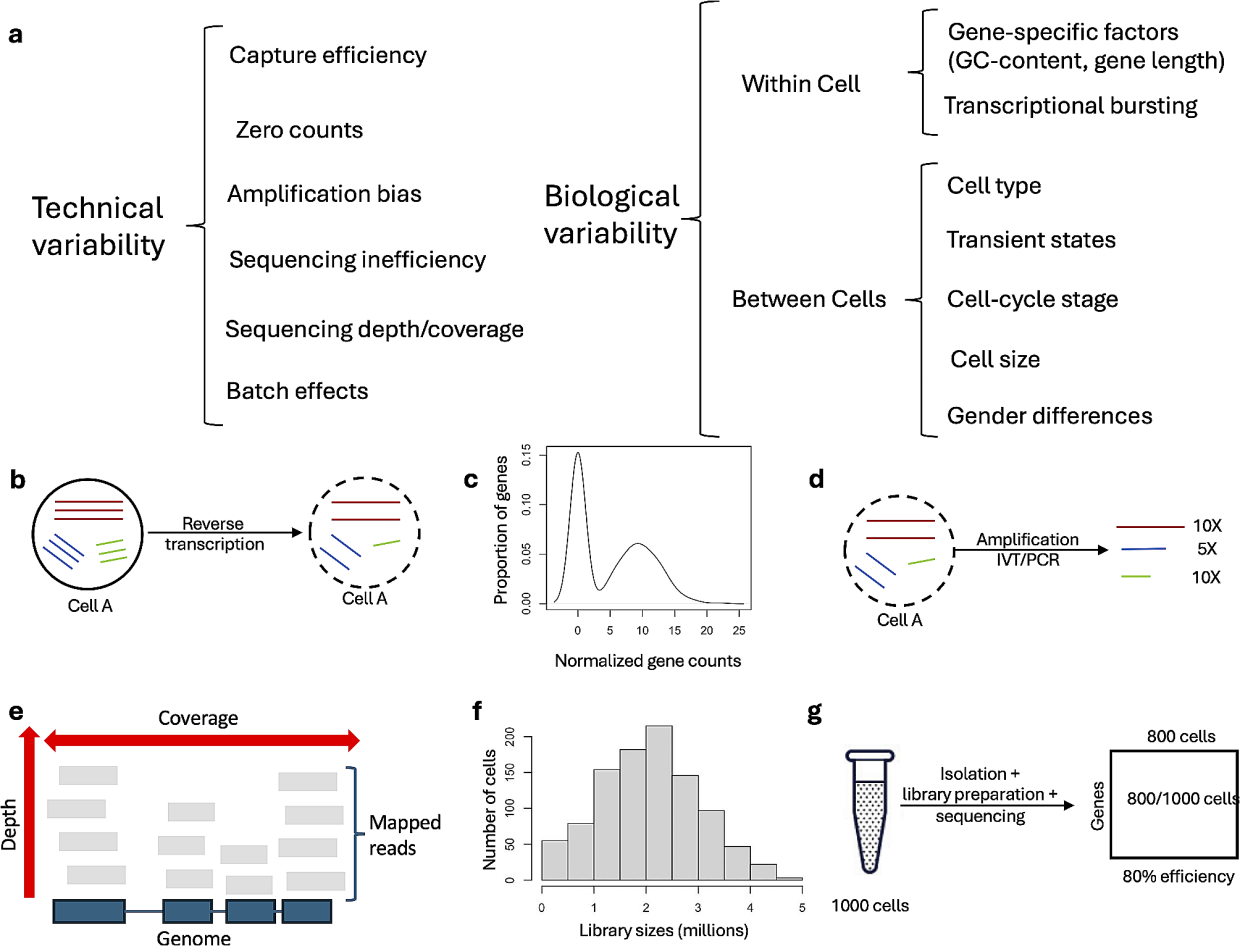
Sources of variability in scRNA-seq datasets. (**a**) Technical and biological variability. (**b**) Capture inefficiency resulting from selection of single-cells and random reverse transcription of mRNA molecules. Colored lines represent different mRNAs. (**c**) Density plot depicting a typical bimodal distribution with a zero inflated behavior representing the number of gene counts across cells. (**d**) Bias observed in IVT or PCR amplification. Certain mRNA molecules are amplified more efficiently than others. (**e**) Sequencing depth and coverage. (**f**) Histogram depicting variable library sizes across cells. (**g**) Sequencing inefficiency showing numerous cells sampled but not sequenced due to errors in measurement

Compared to bulk RNA-seq, scRNA-seq suffers from a high cell-to-cell variability, also referred to as “overdispersion”. The dispersion observed in gene counts of cells from the same type is a combination of two sources of variability, technical and biological (see Fig. 2a). Technical variability or noise is derived from an imperfect measurement process, as is the case of scRNA-seq [40]. Sources of technical variability include capture inefficiency (Fig. 2b), zero counts (Fig. 2c), amplification bias (Fig. 2d), sequencing depth and coverage (Fig. 2e), library size (Fig. 2f), sequencing inefficiency (Fig. 2g), and batch effects. Additionally, individual cell’s read counts depict biological variability due to various factors, for example, transcriptional bursting, cell subpopulation, cell cycle stage, cell size, cell transient stages, and gender differences.

#### Capture inefficiency and zero counts

A typical mammalian cell contains between 50,000 and 300,000 different transcripts with each molecule depicting between 1 and 30 copies per cell [41, 42]. Due to these very low amounts of transcripts per cell, the methods used to capture, reverse transcribe, amplify, and prepare the sequencing libraries, are inefficient in faithfully representing the number of mRNA molecules per gene per cell. For example, after cell lysis, mRNA is converted into the more stable cDNA generally through RT, also known as first strand synthesis. It has been demonstrated that the small concentration of initial mRNA increases the probability of missing transcripts in the RT stage thus, generating “dropout” events [43]. Kharchenko et al. referred to dropouts as events in which a gene appears highly expressed in one cell but not detected in another one, due to inaccuracies in the RT step [43]. In most protocols, RT is initiated from the poly-A tails of mRNAs through oligo-(dT) priming, commonly including overhangs with adapter sequences, cell barcodes and UMIs. Importantly, it has been demonstrated that the efficiency by which the oligo-(dT) primers capture mRNAs is correlated to the length of the poly-A tails [44] which may undergo changes in response to physiological and pathological processes [45]. Thus, the RT process is a source of stochasticity.

Dropout events frequently lead to excessive zeros, one of the most prominent features of scRNA-seq datasets. These are mainly due to the low amounts of starting material, capturing and amplification inefficiencies, and the low sequencing depths which are commonly used. Intriguingly, even deeply sequenced datasets depict up to 50% of expression values with zero counts [46]. Overall, the efficiency of capturing an mRNA molecule, converting it to cDNA, and successfully amplifying it is low and variable, ranging from 10 to 40% [7, 38, 47, 48]. This is why genes that depict a low expression have a high probability of not being detected and becoming a dropout. Thus, scRNA-seq computational methods face the challenge of distinguishing real zero counts from those generated from technical variations (measurement errors) [40].

#### Amplification bias, sequencing depth, coverage, and library size

After RT, second strand synthesis takes place from either a random position or from the end of the first-strand as part of the amplification process. Importantly, both RT and DNA polymerase are processive enzymes that can incorporate large numbers of nucleotides in consecutive reactions before the reaction stops [49, 50]. Consequently, the exact stopping points are unknown. This introduces complex positional dependencies and generates global bias, affecting sequencing coverage [49]. Sequencing depth and coverage are closely related terms referring respectively to the number of times a specific base of the DNA is sequenced and to the proportion of the genome that was sequenced with a certain depth (Fig. 2f). Sequencing depth can be configured as a parameter of the sequencer. A higher sequencing depth may increase coverage at the expense of cost. However, capture inefficiencies and amplification biases have an impact on coverage no matter the sequencing depth, and they need to be corrected.

Given the minimum amount of starting material, the library preparation process requires more than a million-fold amplification [43]. This extensive amplification (either PCR or IVT) leads to additional technical variability, given that some genes may experience preferential amplification [51–53]. Capture inefficiencies and amplification biases generate variable library sizes, defined as the total number of reads per cell. Normalization methods aim at estimating a “library size factor” to correct cell-specific biases related to the number of reads per cell.

The amplification process can also generate dropout events. Thus, UMIs are introduced and they have been reported to substantially reduce unwanted variation due to differences in gene lengths and amplification efficiencies [17]. UMIs are random sequences that are used for tagging cDNA molecules in the 5’ end during RT enabling the accurate quantification of mRNAs by establishing a specific identity for each molecule. Adding UMIs to the reactions before PCR amplification also allows for the bioinformatic identification of PCR duplicates. To date, the majority of scRNA-seq protocols allow transcript UMI-tagging (See Additional file 1).

#### Batch effects

Another important source of technical variation comes with batch effects. Batch effects are common in high-throughput experiments, and they occur when cells from one group or condition are processed (cultured, isolated, prepared library and sequenced) separately (space or time) from cells of another condition [54]. Batch effects also occur when single-cell datasets are compiled from multiple experiments, for example, when integrating large single-cell atlases [55, 56]. In these cases, experiments are most likely performed with different technologies, capturing times, handling personnel, reagents, and equipment. Removing batch effects is a critical and challenging step. Furthermore, studies have demonstrated that batch effects can be highly nonlinear, therefore it is difficult to adjust technical variability without introducing artifacts or confounding real biological variation [54]. Batch effect adjustment methods will be described in the next section.

#### Spike-ins may account for technical variability

An alternative proposed to account for the sources of technical variability described is the use of spike-ins [57]. Spike-ins are non-biological RNA molecules that are added in a fixed concentration to each cell’s lysate and undergo the same processing as endogenous transcripts. In this way, spike-in transcripts are affected by the same inefficient capturing and amplification processes, and after sequencing, the number of spike-in molecules can be compared to the counts obtained and used as a scaling factor for normalization [16, 58, 59]. However, spike-ins can’t easily be incorporated into high throughput cell isolation protocols (e.g. Droplet-based) and in other cases, it is not feasible to consistently add the same quantity of spike-in RNA to every cell [60]. Furthermore, the use of spike-ins has been questioned arguing that synthetic spike-ins behave differently than endogenous transcripts [61].

#### Sources of biological variability

Biological variability is one of the main interests in scRNA-seq and it is the basis of numerous downstream analyses, for example, clustering and differential gene expression. An important complication in addressing biological variability, besides separating it from technical noise, is that gene expression is inherently stochastic. Researchers have demonstrated substantial variability in the amount of mRNA even between genetically identical cells grown under the same conditions [62, 63]. This variability has been explained partially by a stochastic phenomenon known as transcriptional “bursting” [64]. Using gene trap and transgenic cell lines, Suter et al. found that most genes appear to have dynamic fluctuations of expression separated by silent intervals, generating gene-specific temporal transcription patterns [64]. Furthermore, it has been established that gene transcription and protein translation are regulated by combinatorial interactions between molecules undergoing random biochemical reactions [63, 65]. Additionally, the same gene will not be transcribed simultaneously in different cells since individual cells are engaged in dynamic physiological processes, for example, stress response, cell cycle or transient cellular states. Overall, scRNA-seq computational methods must be able to separate the wanted from unwanted variability in datasets characterized by noise (dispersion), abundant zeros, and high-magnitude outliers.

### Normalization methods

An essential first step in the analysis of scRNA-seq data is normalization, whose main aim is to make expression counts comparable within and between cells. Normalization has a strong impact on the detection of differentially expressed genes [66–68] and thus in the number of cell clusters identified. Adequate normalization methods are essential since they underlie the validity of downstream analysis. A normalization pipeline generally includes a combination of imputation, normalization, and batch effect correction processes. However, certain normalization methods, for example, ZIMB-WaVE [69] and Seurat [70] perform all processes.

An early decision in the normalization pipeline selection is whether an imputation method should be included. Recently developed single-cell isolation methods, for example, droplet-based methods yield an incredibly high number of zeros (sometimes exceeding 90%) in the expression matrix [71]. Thus, imputation methods have been proposed. A comprehensive compendium of imputation methods is described by Lähnemann et al. [72]. The main aim of these methods is to predict read counts in cases were experimental or technical noise has led to zero counts, thus generating adjusted data values that better represent true expression. Data smoothing methods, such as Markov Affinity-based Graph Imputation of Cells (MAGIC) [73] detect all zeros as “missing data” and output a matrix with zeros smoothed out. However, the main challenge of these methods is preserving biological zeros. This is especially important in cases where the lack of expression of marker genes is needed to identify a subpopulation of cells [74, 75]. In such cases, the use of model-based or data reconstruction methods that can selectively preserve zeros, for example ALRA [75], SAVER [76], and scImpute [77] is suggested [72].

Some imputation tools use raw scRNA-seq UMI or read counts as input, while others require a normalized count matrix, typically a log-transformation. A log transformation of read counts attempts to reduce the skewness. Researchers have demonstrated that directly processing an expression matrix with a high incidence of zeros may be detrimental for downstream analysis such as clustering and visualization [75]. However, an extensive evaluation found no improvement in the performance of imputation methods against no imputation when comparing clustering and trajectory analysis results [78]. Another study found that some imputation methods introduced false positive signals when identifying differentially expressed genes [74]. Imputation methods are beneficial when the amount of sparsity (biological and technical zeros) is unusually high or when downstream algorithms can’t handle sparse count data [72]. Nevertheless, there is no consensus on the advantages of using imputation algorithms.

Normalization methods are performed after imputation or at the beginning of the pipeline in case imputation was not selected. Broadly, normalization methods can be classified as within and between-sample algorithms according to the correction performed. In the former, counts are adjusted to account for gene-specific features, for example GC-content and gene length, yielding comparable gene expression values within each cell. In the latter, cell-specific features are addressed, for example sequencing depth, resulting in comparable gene expression values across cells [79]. Most methods can use read counts or UMI counts. UMI counts remove amplification biases in non-zero gene count measurements [17]. However, UMIs do not recover sampling zeros. The choice of normalization method (with or without UMIs) is a statistical consideration and is not correlated to the proportion of zeros or the distinction between technical and biological zeros [80].

According to the mathematical model used, normalization approaches can further be classified into global scaling methods, generalized linear models (GLMs), mixed methods, and machine learning-based methods. Additional examples of methods from each category are included in Additional file 2. Furthermore, a compilation of independent benchmarking studies evaluating the performance of normalization methods is found in Additional file 3. Given the importance of normalization methods on the validity of downstream analysis, we will describe common methods belonging to each category. We also discuss batch effect correction methods as the last step in a normalization pipeline.

#### Global scaling methods

Global scaling normalization methods assume that the RNA content is constant for all cells and therefore, a scale factor can be applied to all genes so that there is no difference in expression between cells. These methods are based on the calculation of size factors for each cell to account for differences in library size. For each cell, counts are divided by their corresponding size factors, generating relative abundances. The simplest approach using this assumption is library size normalization, for example transcripts or counts per million (TPM [81], CPM [82]) or reads per kilobase of exon model per million mapped reads (RPKM) [83]. However, these methods are affected by a small proportion of highly expressed genes and can bias differential gene expression results [66]. Normalization methods that address gene length bias, for example TPM and RPKM, are suggested for plate-based full-length sequencing methods. In contrast, droplet-based methods that use UMIs, tag only 3’ or 5’ ends of transcripts and are not affected by gene length [84].

A set of global scaling methods rely on the use of external spike-ins added in a known concentration and processed in parallel with endogenous transcripts. The number of read or UMI counts for spike-in transcripts is then used to scale the counts for each cell, making spike-in gene counts the same across all cells [59]. The caveats of using spike-ins have been previously described. An alternative to spike-in normalization is using a set of genes that have constant expression across cells. These can be housekeeping genes or stably expressed genes. The use of housekeeping genes has been criticized because they may be affected by transcriptional bursting. Lin et al. proposed the ISnorm (Internal Spike-in-like-genes normalization) algorithm to select stably expressed genes based on their pairwise variance and use them to estimate unbiased size factors [85]. A pioneering approach expected to become a gold standard for single-cell RNA counting consists on using molecular spikes [86]. Molecular spikes are RNA spike-ins that contain built-in UMIs enabling the detection, quantification, and correction of artifactual RNA counting even in experiments lacking UMIs. Researchers demonstrated that molecular spikes allow the accurate estimation of total mRNA counts across cells [86].

Other global scaling methods have been adopted from bulk RNA-seq analysis, for example DESeq’s median of ratios [87] and EdgeR’s trimmed mean of M values (TMM) [60]. In DESeq2’s method, a pseudo reference sample is created from the geometric mean of genes across cells, and it is used to generate a sample-specific scaling factor [87, 88]. TMM filters out highly expressed genes as well as those with a large variation and a weighted average of the remaining genes is used to calculate a normalization factor [60, 88]. These methods rely on the assumption that most genes are not differentially expressed. Furthermore, the high frequency of zeros in scRNA-seq datasets may result in nonsensical scaling factors (DESeq2) or undefined M values (TMM) [89].

Most methods implemented for between-sample normalization calculate global scaling factors which are applied to all gene counts of a cell to adjust for sequencing depth. However, these methods fail due to the technical biases described. One of the most common systematic variations observed in scRNA-seq is the unequal relationship between transcript expression and sequencing depth. Global scaling normalization methods can not accurately adjust cell counts in respect to sequencing depth when the ratio is uneven and depends on the expression level. These methods will generate an over-correction for genes with low to moderate expression as well as an under-normalization for highly expressed genes [66]. To circumvent this problem, some global scaling normalization methods rely on pre-clustering (pooling) strategies as will be described.

One of the first normalization methods specifically developed for scRNA-seq was BASiCS (Bayesian Analysis of Single-Cell Sequencing Data) [90]. BASiCS implements an integrated Bayesian hierarchical model to infer cell-specific normalizing constants based on distinguishing technical noise from biological variability [90]. The original implementation of BASiCS relied on the use of spike-ins to estimate technical noise; however, the method was extended to work with multiple independent replicates [91]. It is important to note that BASiCS was designed to be implemented in scenarios where the cell types under study are known a priori, thus unsupervised settings are not recommended (https://github.com/catavallejos/BASiCS). Another highly used scaling-based normalization method is scran. Compared to other methods, scran groups cells with similar library sizes (pre-clustering), estimates a pool-specific factor by summing expression values across pools, and then estimates cell-specific size factors by deconvolving pooled factors [89]. This deconvolution method is implemented in the computeSumFactors function of the scran R package [92].

A study performed by Buttner et al. compared the batch correction performance of 7 global scaling normalization methods including CPM based on library size, relative log expression, TMM, TPM, qsmooth [93], mean ratios, and scran size factor estimation, and demonstrated that scran outperformed other normalization methods [94]. Another benchmarking study assessed the performance of scran, SCnorm, Linnorm, Census, MR, and TMM [95]. Researchers concluded that scran was the best normalization method due to its good performance in common scRNA-seq scenarios with a high number of DEGs and differing levels of mRNA between cells [95]. Interesting results were reported by Ahlmann-Eltze and Huber in a benchmarking study where 22 transformations were applied to UMI-based scRNA-seq datasets [96]. The transformations used had the objective of adjusting UMI counts for variance stabilization, and they included delta method-based, residuals-based, latent gene expression-based, and count-based factor analysis transformations. The best performing transformation was the logarithm with a pseudo-count followed by PCA according to k-nearest neighbor (k-NN) based metrics.

A major caveat of global scaling factor normalization methods is that they assume that RNA content is constant for all cells and use the same scaling factor for all genes. Therefore, alternative normalization methods have been proposed.

#### Generalized linear models

Initial comparisons of the expression of genes between cells of the same type demonstrated that they were lognormally [97] or Gamma distributed [98]. Others have suggested that models of gene expression should incorporate the thermodynamic contribution to technical noise, which follows a Poisson distribution [99]. Mixed Poisson distributions have been widely used to model non-homogeneous scRNA-seq datasets, for example Beta-Poisson [100] and Gamma-Poisson [101–104]. The implementations of these models can be extended to allow variations between cells using GLMs.

GLMs are a statistical tool used to model the contribution of systematic and random components to a response variable (gene or UMI counts). GLMs include classical linear regression models and count-based models. Furthermore, GLMs allow the modeler to express a relationship between covariates, that will be regressed out, and a response variable in a linear, additive manner [105]. In this sense, covariates account for unwanted technical variability, for example sequencing depth, while biological variability is captured in the response variable. A commonly used regression model is Linnorm, a linear model and normality-based transformation method. Linnorm calculates normalization and transformation parameters based on stably expressed genes across different cells and fits the log-transformed expression data to a linear model [106]. Other common regression-based normalization approaches are PsiNorm and SCnorm. PsiNorm performs normalization between samples by fitting data into a Pareto power-law distribution providing comparable performance as scran and Linnorm with shorter runtime and memory efficiency [107]. SCnorm first performs a quantile regression for every gene to determine the dependence of gene-specific expression on sequencing depth, and then a second quantile regression estimates scale factors for groups of genes [108]. A benchmarking study systematically compared the performance of combinations of methods for normalization and imputation, clustering, trajectory analysis, and data integration [109]. Authors evaluated 8 popular normalization methods including BASiCS, scran, SCnorm, and Linnorm using mixtures of cells or RNA by calculating the silhouette width of clusters and the Pearson correlation coefficient of normalized gene expression. This pioneering mixology experiment demonstrated that scran and Linnorm had consistent satisfactory results and Linnorm’s performance was invariant to the input dataset [109].

GLMs have also been proposed to model read counts using probability distributions. Commonly used count distributions to model gene counts across single-cells include non-zero inflated: Poisson and negative binomial (NB), and zero-inflated: Poisson (ZIP) and NB (ZINB). These methods have slight differences in how they calculate the probability of zero counts. Poisson methods have only one parameter, $\lambda$ corresponding to mean and variance. The assumption of Poisson normalization methods is that the frequency of a given transcript is uniform across cells and variation is derived from independent statistical sampling. However, as previously explained, variations in counts are rooted in both technical and biological factors, making the use of this distribution inappropriate. ZIP and NB incorporate an additional parameter each ($p, \psi$) to model the proportion of non-Poisson zeros and overdispersion of variance relative to the mean respectively, whereas ZINB incorporates both. It has been demonstrated that the sampling distribution of UMI counts (plate-based or droplet-based) is not zero inflated, as compared to read counts [104, 110]. Thus, if UMIs are used, normalization methods involving zero inflation are not appropriate [104, 111, 112]. NB provides a better approximation to model UMI count data [113]. It assumes random transcript frequencies and includes a parameter to quantify overdispersion. NB regression models account for cell-specific covariates, for example sequencing depth [101]. However, researchers have demonstrated that modeling single-cell data with a NB distribution may lead to overfitting [102]. By comparing these four distributions (Poisson, NB, ZIP, ZINB) using the same mean, Jiang et al. showed that ZINB depicts the highest proportion of zeros (∼ 64%) whereas NB and ZINB depict bigger probabilities of finding larger values [114].

Variations of count-based GLMs have been proposed. Hafemeister et al. developed scTransform, a regularized NB regression in which UMI-based gene counts are the response variable and sequencing depth is a covariable [102]. The Pearson residuals from this regularized NB regression accurately represent the normalized data values and can be used as an input to dimensionality reduction algorithms. scTransform v2 effectively performs variance stabilization and performs better than others for variable gene identification and differential expression analysis [115]. It is available as an R package and can be used through Seurat toolkit. Researchers also modelled gene counts per cell as a random variable following a zero-inflated NB (ZINB) distribution however, allowing the inclusion of cell and gene level covariates [69]. This method was named ZIMB-based Wanted Variation Extraction (ZIMB-WaVE) [69]. Covariates are introduced as parameters in regression equations, and they are inferred through a penalized maximum likelihood procedure. Interestingly, this method can also be used for dimensionality reduction. In another approach, a Gamma Regression Model (GRM) was proposed to reduce the noise in scRNA-seq data [116]. GRM relies on spike-ins to train a model that fits a GRM between sequencing reads and spike-in concentrations.

#### Mixed methods

In mixed methods, normalization is performed through the combined implementation of different approaches. Mixed methods are very important in addressing the characteristic bimodal expression pattern of single cells, where abundant genes appear to either have a high expression or to be undetected. These methods can model various sources of technical variability independently using different probability distributions for each. One of the first approaches in using this class of normalization was single cell differential expression (SCDE) proposed by Kharchenko et al. [43]. SCDE models cell counts as a mixture of two probabilistic processes: a negative binomial corresponding to normal gene amplification and detection, and a Poisson distribution accounting for zero counts. The optimal parameters corresponding to each distribution are then determined through a multinomial logistic regression. SCDE is implemented in the pathway and gene set overdispersion analysis (PAGODA) in which cell-specific error models are used to estimate residual gene expression variance allowing the identification of pathways and gene sets depicting significant coordinated variability [117].

Similarly, the “Model-based Analysis of Single-cell Transcriptomics” (MAST) [118], uses a hurdle model implemented as a two-part generalized linear model that simultaneously models the fraction of genes that are detectably expressed in each cell (cellular detection rate: CDR) and the positive gene expression values. MAST models gene expression rate using a logistic regression and a Gaussian distribution is used to model the expression level depending on a gene being expressed in a specific cell. MAST is available as an R library in Bioconductor, and it includes functions for cell filtering, adaptive noise thresholding, univariate differential gene expression with covariate adjustment, gene-gene correlations and co-expression, and gene set enrichment analysis.

#### Deep learning-based methods

Deep learning, a subclass of machine learning, has been recently used to analyze high-throughput omics data, including scRNA-seq [119]. Deep learning consists of neural network architectures to discover latent and informative patterns in complex data incorporating thousands of trainable parameters and finds transformations that can effectively normalize counts preserving biological information [120]. Deep learning approaches for scRNA-seq data normalization include autoencoders, variational autoencoders, and graph neural networks [121]. Variational autoencoders are a popular class of unsupervised learning methods. For example, single cell variational inference (scVI) learns cell-specific scaling factors by modeling the expression of a gene in a cell as a sample from a ZINB distribution incorporating a batch annotation of each cell and two unobserved random variables [122]. Deep learning methods proposed for scRNA-seq data analysis have been reviewed by Brendel et al. [119]. While these emerging methods are promising, independent benchmarking studies comparing their performance against traditional statistical methods are needed.

#### Batch effect correction methods

Batch effect correction methods aim at removing technical variability derived from experimental design without altering biological variability. Technical variability is systematic, and it is introduced from multiple sources, as previously described. This variability can be confounded as biological and thus, its removal is essential. Methods developed for microarray and bulk RNA-seq data batch correction such as ComBat [123] and limma [124] have been used. These methods use a linear regression to model the relationship between batch and gene expression. Other methods, for example ZINB-WaVE extend the linear model based on a zero-inflated negative binomial distribution, accounting for data sparsity, over-dispersion, and non-linear batch effects [69]. A caveat of linear regression methods is that they assume that the composition of cell subpopulations is identical from batch to batch, making them prone to overcorrection [54]. However, in scRNA-seq, subpopulation composition is not the same across batches. Therefore, methods relying on the identification of shared cell types across batches have been developed, for example mutual nearest neighbors (MNN) [125]. This method identifies cells with similar expression profiles between two batches and then estimates a correction vector using the mean differences in gene expression between cells in MNN pairs. The correction vector is then used to align datasets in a shared space, eliminating batch effects. Since the MNN search is performed in a high dimensional space, this method’s caveat is a high memory consumption and CPU runtime. To overcome this problem, numerous algorithms have been developed with the characteristic that the nearest neighbor search is performed in a common reduced dimensional embedding using for example PCA [126], canonical correlation analysis (CCA) [127], non-negative matrix factorization (NMF) [128], and singular value decomposition (SVD) [129]. Common examples of these methods include fastMNN [125] and Harmony [130] which use PCA, Seurat MultiCCA [70] that captures correlated pairs in a CCA dimensionally reduced space, LIGER [131] which uses integrative NMF to transform data into a low-dimensional space, and Scanorama [132] that implements SVD for neighbor search. These unsupervised methods based on MNN may incorrectly match neighboring cells from different clusters across batches, leading to spurious results.

Supervised MNN methods have also been proposed, for example SMNN [133] and iSMNN [134]. These methods require the same cell type across batches since they incorporate cell-type specific information to restrict the detection of MNNs. Cell type labels across shared cells in all batches are determined through prior knowledge or inferred by an unsupervised clustering approach. Deep learning-based methods have also become popular for batch effect correction. For example, deepMNN [135] attempts to remove batch effects using a residual neural network that minimizes batch loss, defined as the sum of the Euclidean distances between MNN pairs in PCA space. However, most of the methods based on MNN only analyze two batches at a time, introducing a batch correction order bias. Furthermore, most of these algorithms remove batch effect and then cluster cells, increasing the probability of missing rare cell types. To solve these issues, the batch alignment of single cell transcriptomics data using a deep metric learning (scDML) model has recently been proposed [136]. scDML uses deep metric learning to remove batch effects, guided by the initial clusters and MNN information within and between batches.

### Normalization performance assessment

Given the prevalence of confounding factors in single-cell experiments, the lack of gold-standard normalization methods and the ambiguity in selecting parameters used in such methods, a set of metrics and guidelines have been proposed to aid in the selection of the most suitable normalization method. Pilot experiments must be performed to evaluate and compare normalization pipelines. A list of benchmarking studies and the metrics used for evaluating normalization methods is included in Additional file 3. Popular evaluation metrics are described next.

#### Silhouette width

The silhouette width is an established metric used to determine clustering validity [137]. However, it has also been used to compare the performance of normalization methods [94, 109]. In this method, a silhouette width value is calculated for each cluster using the normalized average distance between its cells to cells belonging to other clusters. The first two or three principal components (PCs) of normalized counts are generally used to calculate the Euclidean distances between cells. Larger silhouette widths correspond to a better separation between clusters. A known mixture of cells should be used to identify the best performing normalization method according to the experimental conditions.

#### K-nearest neighbor batch effect test

Performance can also be evaluated through the K-nearest neighbor batch-effect test (kBET) and a PC regression [94]. These two methods are used to evaluate batch effect correction methods. However, authors have tested these methods by sequencing two technical replicates of the same cell type and introducing a known batch effect. Then, data sets have been processed with combinations of imputation, normalization, and batch effect correction methods to determine which pipeline better removes the batch effect preserving biological variability. The kBET relies on the assumption that in a well-mixed replicated experiment, subsets of a fixed number of neighboring cells have the same distribution of batch labels as the complete dataset. To compare batch label distributions, a Pearson’s ${\chi }^{2}$ test is suggested, and a rejection rate is calculated. Intuitively, lower rejection rates are obtained when batch effects have been properly removed and the normalization method is adequate. Alternatively, the scaled explained variance of all PCs significantly correlated with batch effect may also be used to evaluate normalization method performance. In this method, the variance explained by the top 50 PCs is used as a scaling factor. Furthermore, a linear regression between the loadings of each PC and the batch covariate is used to determine a PC’s significance. The amount of scaled variance explained is correlated with the degree of batch effect present in the dataset.

#### Highly variable genes

**Fig. 3:**
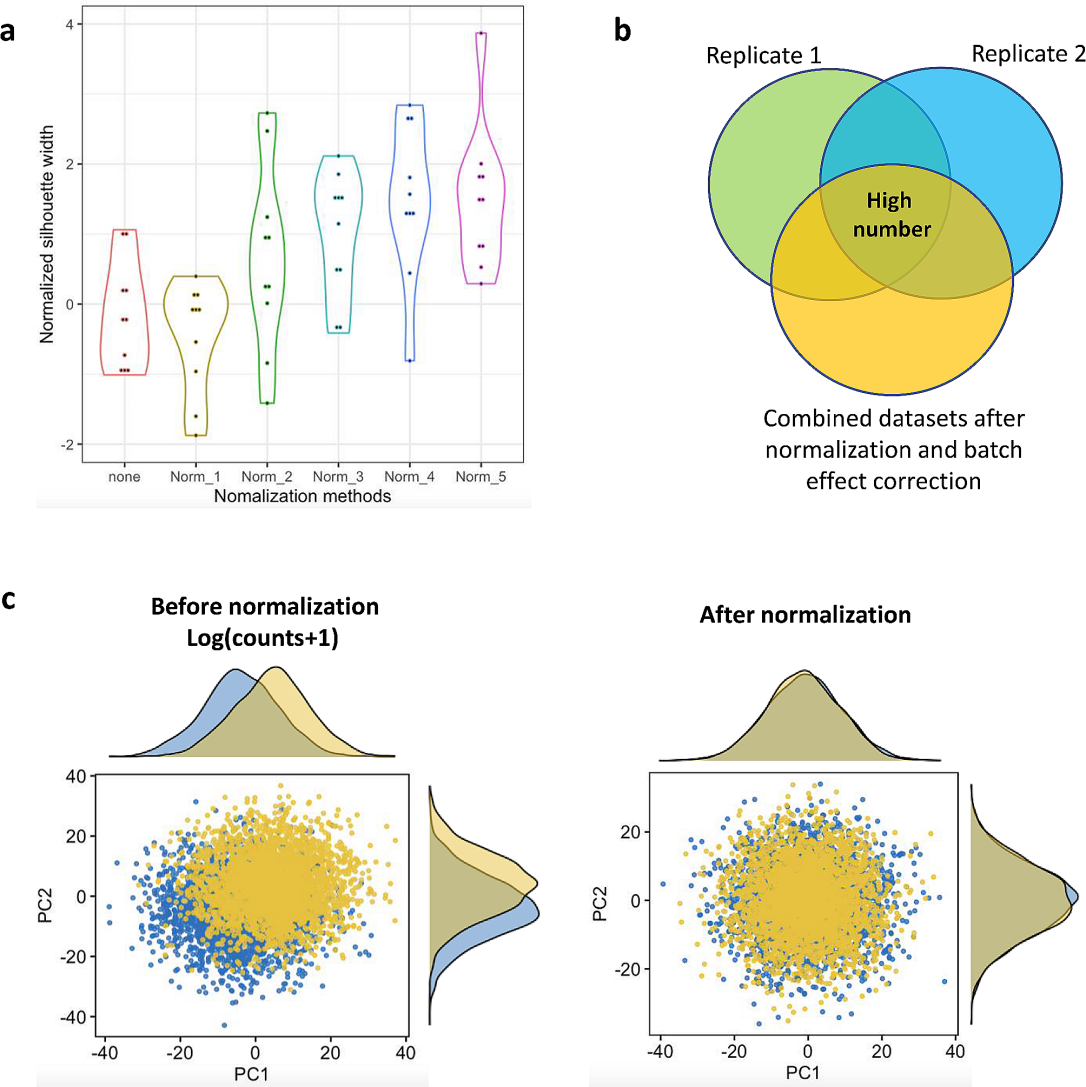
Data-driven metrics used to assess the performance of normalization methods. (**a**) Violin plots depicting the normalized silhouette width obtained by different normalization methods. Larger silhouette widths correspond to a better separation between clusters and thus a better normalization. (**b**) HVGs are identified independently from the raw replicates and the normalized combined datasets. The better normalization performing pipeline will depict the number of HVGs in the intersection of all datasets. (**c**) Schematic representation of the scaled explained variance obtained from the two principal components before and after normalization. Counts in the scenario before normalization were log-transformed

Biological heterogeneity in the datasets should be conserved even after the implementation of imputation, normalization, and/or batch-effect correction methods. By comparing Highly Variable Genes (HVG) before and after normalization pipelines, scientists may determine if biological heterogeneity was preserved [57, 94]. The variability of a gene is obtained through the squared coefficient of variation (CV^2^) of normalized read counts across cells. HVGs are those whose variation is greater than a fixed threshold and they account for the heterogeneity between cells. HVGs should be maintained after a implementing a normalization pipeline and no new HVGs should be introduced. A schematic representation of silhouette width, kBET, scaled explained variance, and HGV metrics for evaluating normalization pipelines is depicted in Fig. 3. Plots such as t-Distributed Stochastic Neighbor Embedding (t-SNE) [138] and Uniform Manifold Approximation and Projection (UMAP) [139] are generally used to visualize cell clusters before and after a normalization pipeline.

#### Scone, a tool for systematic comparison of normalization pipelines

An important tool, Scone, was recently developed by Cole et al. for the comparison of normalization pipelines [140]. Scone is a flexible and modular framework for preprocessing scRNA-seq datasets using multiple normalization strategies and systematically evaluating them through a panel of data-driven metrics. Interestingly, scone evaluates the performance of a range of normalization pipelines and ranks them according to performance metrics, including for example silhouette width. Moreover, scone can incorporate a user-defined normalization pipeline.

### Toolkits

Recently, over 1000 tools for analyzing scRNA-seq data have been developed [141, 142]. Based on the procedure, Zappia et al., separate single-cell data analysis into four analysis phases: data acquisition, data cleaning, cell assignment, and gene identification [142]. The majority of these tools are developed in R or python, and more and more of them will be designed in python in the future [141]. Here, we introduce some toolkits which can perform complete analysis of scRNA-seq datasets (Additional file 4).

Seurat is widely used by researchers, and it starts from a gene expression matrix (read counts) (https://satijalab.org/seurat/). It can compare scRNA-seq datasets from different conditions, technologies, or species. Seurat has two main normalization methods (LogNormalize [143] and sctransform [102]). For the integration of different scRNA-seq datasets, Seurat has two methods (CCA (canonical correlation analysis) [143] and RPCA (reciprocal PCA)) [144] to remove the batch effect. RPCA is an optimization for large numbers of samples and cells [144]. Seurat can provide the clusters from all cells, the expression of marker genes, and differential expression genes among the clusters. Furthermore, Seurat results can be transferred to other platforms or pipelines, for example, Monocle’s pseudotime analysis [145–147], RNA velocity analysis [148], single cell regulation network analysis (SCENIC) [149], and cell-cell communication analysis (e.g., CellChat [150]).

SCANPY is another similar toolkit for scRNA-seq analysis [151]. It is a Python-based tool that starts from a gene expression matrix. It integrates many scRNA-seq analysis methods, such as gene/cell preprocessing, clustering, pseudotime and trajectory inference, and other analysis. The normalization of SCANPY is only based on library size. SCANPY can use four algorithms to remove batch variations, e.g., Regress_Out [151], ComBat [123], Scanorama [132] and MNN_Correct [125, 152]. Compared with R-based Seurat, SCANPY based on Python will have more processing efficiency and running speed [152]. SCANPY has integrated PAGA [153] in the toolkits, so it can directly perform the trajectory analysis.

The use of these toolkits requires programming experience. With the development of scRNA-seq data analysis, some graphical user interfaces analysis tools have also been developed, such as SCorange [154], SCTK (Single Cell Toolkit) [155], Granatum [156], and ASAP (Automated Single-cell Analysis Pipeline) [157]. These web-based analysis tools integrate several normalizations and batch-effect removing methods. For example, Granatum has four normalization methods (e.g., quantile normalization, geometric mean normalization, size-factor normalization, and Voom) and two batch-effect removing methods (e.g., ComBat and Median alignment) [156]. SCTK is built in singleCellTK R package, however, SCTK could analyze sc/snRNA-seq data with graphical user interface (https://sctk.bu.edu/) by Shiny APP [155]. It includes several normalization methods from Seurat (e.g., LogNormalize, Sctransform) and Scater (e.g., CPM, LogNormCounts), and batch-effect removing methods (e.g., MNN, scMerge, Scanorama, and ComBatsSeq).

## Conclusions

Major advances in single cell sequencing technologies have greatly improved our understanding of the complexity of organs and tissues and the dynamism of biological processes. However, a critical step in scRNA-seq data analysis is normalization, a process that aims at making gene counts comparable within and between cells, and among biological replicates. Recent pioneering work by Choudhary and Satija demonstrated that the degree of overdispersion within 59 scRNA-seq datasets varied widely across datasets, systems, and gene abundances, suggesting that the estimation of parameters is dataset-specific [115]. Thus, the selection of a normalization method is not trivial, and it has a direct impact on downstream analysis. For example, a study by Squair et al. [158] found that the most frequently used methods for differential expression analysis (including each methods’ normalization) identified differentially expressed genes even when biological differences were absent. Authors demonstrated a systematic tendency of single-cell methods to identify highly expressed unchanged genes as differentially expressed. Moreover, false differentially expressed genes will affect clustering and trajectory analysis. These results underscore the importance of selecting normalization methods that adequately account for technical noise and variability between biological replicates. Furthermore, another intriguing observation demonstrated by benchmarking studies is that normalization methods perform differently depending on the input dataset. This is likely due to differences in technical noise sources and to the heterogeneity of samples. Instead of comparing the normalization performance on numerous real world or simulated datasets, benchmarking studies should use well designed mixture control experiments as previously proposed [109].

In scRNA-seq count data, cell-to-cell biological variation is related to cell type and state and is encoded in cellular transcriptomes. This heterogeneity is the main source of interest, and it should be modeled to include covariates that influence gene expression. To account for these sources of technical variability, normalization methods depict different approaches. Global normalization methods estimate a size factor for each cell to account for differences in library size. Since the size factor is applied to all genes of a cell, biological variability may be affected. Global scaling normalization methods that rely on pre-clustering or pooling cells with similar library sizes and estimating a pool-specific factor, for example scran, perform better as demonstrated by benchmarking studies [94, 95]. In contrast, generalized linear models use probability distributions to model the contribution of systematic and random components to a response variable, corresponding to gene counts. In this way, covariables account for technical variation, such a sequencing depth, and they are regressed out while the true biological variability is expected to be captured in the response variable. Mixed methods extend linear models by addressing each technical variability source with an independent probability distribution or error model. Emerging deep learning-based methods use neural network architectures to learn underlying patterns of gene expression with complex and non-linear relationships. These methods can efficiently model technical variation sources including batch effects and find optimal transformations that can normalize counts preserving biological variability. Studies using mixture control experiments for benchmarking deep learning-based normalization methods are still needed.

The selection of the most appropriate normalization method is strongly dependent on the experimental design, protocol and platform, and assumptions regarding technical and biological variability need to be made. Thus, there is no better performing normalization pipeline. Instead, pilot experiments should be made to evaluate the performance of a series of normalization pipelines using recommended metrics. These experiments should closely resemble the final experiment, for instance, the same experimental platform and sequencing technology should be used. The selection of the better suited normalization method may be performed through the assessment of data-driven metrics described herein. Moreover, the use of frameworks such as Scone are also recommended to simultaneously evaluate the performance of numerous normalization pipelines.

Further work is needed to develop new tools that perform accurate diagnostics concerning the validity of statistical assumptions under the observed data. Novel approximations such as the introduction of molecular spikes for more accurate molecule counting have the potential of becoming a gold-standard and reducing the technical variability, facilitating the selection of a normalization method.

### Electronic supplementary material

Below is the link to the electronic supplementary material.

Supplementary Material 1

Supplementary Material 2

Supplementary Material 3

Supplementary Material 4

## Data Availability

There are no new data associated with this article.

## Abbreviations

scRNA-seq: single-cell RNA-sequencing
FACS/MACS: fluorescence/magnetic-activated cell sorting
IFCs: integrated fluidics circuits
RT: reverse transcription
ERCCs: External RNA Control Consortium
UMI: unique molecule identifier
IVT: in vitro transcription
TSO: template-switching oligonucleotides
STRT-seq: single-cell tagged reverse transcription sequencing
Smart-seq: switching mechanisms at the 5’-end of the RNA transcript sequencing
CEL-seq: cell expression by linear amplification and sequencing
MARS-seq: massively parallel single-cell RNA sequencing
inDrops-seq: indexing droplets RNA sequencing
GLMs: generalized linear models
TPM/CPM: transcripts or counts per million
RPKM: reads per kilobase of exon model per million mapped reads
ISnorm: Internal Spike-in-like-genes normalization
TMM: trimmed mean of M values
BASiCS: Bayesian Analysis of Single-Cell Sequencing Data
ZINB: zero-inflated NB
ZIMB-WaVE: ZIMB-based Wanted Variation Extraction
GRM: Gamma Regression Model
SCDE: single cell differential expression
PAGODA: pathway and gene set overdispersion analysis
MAST: Model-based Analysis of Single-cell Transcriptomics
MNN: mutual nearest neighbors
CCA: canonical correlation analysis
NMF: non-negative matrix factorization
SVD: singular value decomposition
PCs: principal components
kBET: K-nearest neighbor batch-effect test
t-SNE: t-Distributed Stochastic Neighbor Embedding
HVG: Highly Variable Genes
UMAP: Uniform Manifold Approximation and Projection
RPCA: reciprocal PCA
SCENIC: single cell regulation network analysis
SCTK: Single Cell Toolkit
ASAP: Automated Single-cell Analysis Pipeline
PCR: polymerase chain reaction
KOAc: potassium acetate
MgOAc: magnesium acetate
NA: not available
